# Naa20, the catalytic subunit of NatB complex, contributes to hepatocellular carcinoma by regulating the LKB1–AMPK–mTOR axis

**DOI:** 10.1038/s12276-020-00525-3

**Published:** 2020-11-20

**Authors:** Taek-Yeol Jung, Jae-Eun Ryu, Mi-Mi Jang, Soh-Yeon Lee, Gyu-Rin Jin, Chan-Woo Kim, Chae-Young Lee, Hyelee Kim, EungHan Kim, Sera Park, Seonjeong Lee, Cheolju Lee, Wankyu Kim, TaeSoo Kim, Soo-Young Lee, Bong-Gun Ju, Hyun-Seok Kim

**Affiliations:** 1 Department of Life Science, College of Natural Science, Ewha Womans University, Seoul, 03760 South Korea; 2 Department of Life Science, College of Natural Science, Sogang University, Seoul, 04107 South Korea; 3 Department of Biochemistry, College of Medicine, The Catholic University of Korea, Seoul, 06591 South Korea; 4 Department of Biochemistry, College of Natural Science, Chungbuk National University, Cheongju, 28644 South Korea; 5 KaiPharm, Seoul, 03759 Republic of Korea; 6 Center for Theragnosis, Korea Institute of Science and Technology, Seoul, 02792 South Korea; 7 Division of Bio-Medical Science and Technology, KIST School, Korea University of Science and Technology, Seoul, 02792 South Korea; 8 Department of Converging Science and Technology, KHU-KIST, Kyung Hee University, Seoul, 02447 South Korea; 9 The Research Center for Cellular Homeostasis, Ewha Womans University, Seoul, 03760 South Korea; 10 Department of Bioinspired Science, Ewha Womans University, Seoul, 03760 South Korea; 11 The Fluorescence Core Imaging Center, Ewha Womans University, Seoul, 03760 South Korea

**Keywords:** Cancer prevention, Tumour biomarkers

## Abstract

N-α-acetyltransferase 20 (Naa20), which is a catalytic subunit of the N-terminal acetyltransferase B (NatB) complex, has recently been reported to be implicated in hepatocellular carcinoma (HCC) progression and autophagy, but the underlying mechanism remains unclear. Here, we report that based on bioinformatic analysis of Gene Expression Omnibus and The Cancer Genome Atlas data sets, Naa20 expression is much higher in HCC tumors than in normal tissues, promoting oncogenic properties in HCC cells. Mechanistically, Naa20 inhibits the activity of AMP-activated protein kinase (AMPK) to promote the mammalian target of rapamycin signaling pathway, which contributes to cell proliferation, as well as autophagy, through its N-terminal acetyltransferase (NAT) activity. We further show that liver kinase B1 (LKB1), a major regulator of AMPK activity, can be N-terminally acetylated by NatB in vitro, but also probably by NatB and/or other members of the NAT family in vivo, which may have a negative effect on AMPK activity through downregulation of LKB1 phosphorylation at S428. Indeed, p-LKB1 (S428) and p-AMPK levels are enhanced in Naa20-deficient cells, as well as in cells expressing the nonacetylated LKB1-MPE mutant; moreover, importantly, LKB1 deficiency reverses the molecular and cellular events driven by Naa20 knockdown. Taken together, our findings suggest that N-terminal acetylation of LKB1 by Naa20 may inhibit the LKB1–AMPK signaling pathway, which contributes to tumorigenesis and autophagy in HCC.

## Cancer: A switch that regulates liver tumor formation

Chemical modifications convert a protein that normally acts as a tumor suppressor into a driver of liver cancer cell proliferation. Many proteins are regulated via enzymatic addition of chemical groups. Researchers led by Hyun-Seok Kim at Ewha Womans University in Seoul, South Korea, set out to characterize the function of NatB, one of N-terminal acetyltransferas family that adds acetyl chemical groups to the first amino acid on selected target proteins. They found that mutations that disable NatB inhibits growth of hepatocellular carcinoma cells. Subsequent experiments provided evidence that NatB modifies a signaling protein called LKB1, which has previously been identified as a safeguard against cancer. Loss of LKB1 modification has downstream effects that favor tumor formation. These results suggest that drugs targeting NatB function may be beneficial in certain cancer subtypes.

## Introduction

N-terminal acetylation (Nt-acetylation) is one of the most prevalent cotranslational modifications of eukaryotic proteins, although some recent studies have shown that it also occurs posttranslationally^1^. This reaction is catalyzed by a set of enzyme complexes called N-terminal acetyltransferases (NATs), which have recently been reported to be composed of seven NAT complexes (NatA–NatH) in eukaryotes^1–3^. Each NAT complex seems to be specific for the first and second N-terminal amino acid residues of the nascent protein^1^. Among these complexes, N-terminal acetyltransferase B (NatB), which is composed of the catalytic subunit N-α-acetyltransferase 20 (Naa20) and the auxiliary subunit Naa25, exhibits strong preferences for proteins starting with a methionine-acidic/hydrophilic amino acid motif at their N-termini (i.e., MD-, MN-, ME-, and MQ-)^1^. Thus, NatB can presumably acetylate the N-terminus of 15% and 18% of all yeast and human proteins, respectively^4^; however, a recent study identified only 180 human and 110 yeast NatB substrates through a combined quantitative N-terminomic approach^4^, probably indicating the existence of substrate redundancy among NATs.

Accumulating studies have reported that Nt-acetylation is implicated in a wide range of pathological processes, including tumorigenesis, developmental defects, and neurodegeneration^1–3^. Notably, many NATs may play an oncogenic role in tumorigenesis in diverse cancers^1–3,5^. At the protein level, Nt-acetylation has been discovered to regulate various protein activities, such as degradation, protein–protein interaction, and localization^1–3^. For the more typical NAT, which Nt-acetylates many thousands of proteins, it can be challenging to connect effects at the protein level to specific cellular and organismal phenotypes.

In yeast, the naa20-Δ and naa25-Δ (mdm20-Δ) deletion mutants exhibited a variety of cellular defects, including reduced mating; aberrant morphology; defective mitochondrial division and vacuolar segregation; elevated sensitivity in response to several stresses, such as high temperature, caffeine, and DNA damage; and, most recently, abnormal NAD^+^ homeostasis^6–9^. According to previous reports, some of these defects in yeast may have resulted primarily from abnormal cytoskeletal functions caused by the lack of Nt-acetylation in either or both of two critical cytoskeletal proteins—actin and tropomyosin—or in an unknown protein^7,8^. In addition, Nt-acetylation of nicotinamide mononucleotide adenyltransferases (Nma1 and Nma2) was reported to be essential for maintaining NAD^+^ homeostasis in yeast^9,10^. In mammals, NatB has been shown to participate in several cellular processes, including growth^11,12^, autophagy^13–15^, and viral infection^16^, by altering the levels of cell cycle-related genes, mammalian target of rapamycin (mTOR)C2 signaling, and the hippo/YAP and ERK1/2 pathways^11–18^, respectively, but the underlying mechanisms connecting Nt-acetylation with proteins are still unknown. Regarding tumorigenesis, several studies revealed that both subunits of NatB are upregulated in hepatocellular carcinoma (HCC) tumor tissues compared with nontumor tissues^11,12^, suggesting that NatB may be implicated in promoting tumorigenesis. Moreover, silencing Naa20 or Naa25 in HCC cells leads to dysfunction of cyclin-dependent kinase 2 or tropomyosin, and to subsequent impairment of several proliferative signals or pathways, resulting in significant growth retardation^11,12^. However, the underlying mechanisms by which NatB-mediated Nt-acetylation affects cell proliferation need to be further elucidated.

It has been well documented that liver kinase B1 (LKB1) regulates various cellular processes, such as metabolism, proliferation, and migration, by phosphorylating and activating several kinases, including AMP-activated protein kinase (AMPK)^19^. Moreover, mutation and dysregulation of LKB1 have been reported to occur in most types of tumors, and LKB1 is thus considered a tumor suppressor in a wide variety of organs^19,20^. However, some recent studies have revealed that LKB1 is upregulated in animal models of HCC and in tumor tissues of HCC patients^20–22^, indicating that it may have a dual role in tumorigenesis. LKB1 forms a complex with the pseudokinase ste20-related adaptor (STRADα) and the scaffolding protein mouse protein 25 (MO25), which induces the cytoplasmic localization and promotes the activity of LKB1 (refs. ^19,20^). In addition, accumulating studies have revealed that the activity of LKB1 is also controlled by several types of posttranslational modifications, such as phosphorylation, lysine acetylation, ubiquitination, and methylation^20^. Thus, it has been proposed that the comprehensive PTMs of LKB1 in HCC may contribute to the dual role of LKB1 in tumorigenesis.

Interestingly, one previous study showed that naa20 deletion in yeast caused elevated phosphorylation levels, and the kinase Snf1p, which is the yeast homolog of AMPK, was predicted to be responsible^23^. This report provided insight into how Naa20 depletion in mammalian cells causes autophagy activation and cell growth retardation, because AMPK is a well-known major regulator of autophagy and cell growth^24^. Considering this assumption, we investigated whether AMPK is responsible for the autophagy activation and growth delay caused by Naa20 depletion in HCC cell lines. Consistent with previous reports, we found that Naa20 silencing led to significant growth retardation and increased autophagy in several HCC cell lines. Importantly, Naa20 negatively regulated the LKB1–AMPK axis to promote the mTOR signaling pathway through Nt-acetylation of LKB1, which contributes to tumor progression and autophagy in HCC. Our results indicate that Nt-acetylation by Naa20 is implicated in the regulation of the LKB1–AMPK–mTOR signaling pathway, which may impact tumorigenesis and autophagy in HCC.

## Materials and methods

### Cell culture

SK-Hep1, Hep3B, and HepG2 cells were purchased from the American Type Culture Collection (ATCC, VA, USA) and maintained under the conditions recommended by the supplier. The Hep3B-GFP-LC3 stable cell line was kindly provided by Professor Yong-Keun Jung (Seoul National University, Korea). All cells were cultured at 37 °C in humidified air with 5% CO_2_ in Dulbecco’s modified Eagle’s medium (DMEM) or Roswell Park Memorial Institute (RPMI) medium (Corning Incorporated, Corning, NY, USA) containing 10% fetal bovine serum (Corning Incorporated, Corning, NY, USA) and 1% penicillin–streptomycin (Corning Incorporated, Corning, NY, USA). The stable cell lines were maintained in DMEM or RPMI medium containing puromycin (2–6 μg/mL).

### Lentivirus production and generation of stable cell lines

For lentivirus production, the lentiviral vector pLKO.1 containing sh-Naa20 (Sigma-Aldrich, St. Louis, MO, USA) was cotransfected with packaging vectors into 293 T cells using Lipofectamine® 3000 (Invitrogen, Thermo Fisher Scientific, Carlsbad, CA, USA). Transfected HEK293T cells were incubated at 37 °C for 24 h, and the medium was then replaced with fresh medium. The supernatant was harvested after incubation for 24 h and filtered through a 0.45-μm filter. To generate stable cell lines, cells were treated with 6 μg/mL polybrene and infected with sh-Naa20-expressing lentivirus for 48 h, after which stable cells were subsequently selected using puromycin (2–6 μg/mL).

### Plasmid and siRNA transfection

Cells were cultured in six-well plates (2 × 10^5^ cells/well). When the cells reached 60% confluence, V5-Naa20 WT, V5-Naa20 YF (Y123F), and pCDH-Naa20 were transfected into the SK-Hep1 and Hep3B cell lines, using Lipofectamine® 3000 (Invitrogen, Thermo Fisher Scientific, Carlsbad, CA, USA). Cells were harvested 48 h after transfection. For transient transfection of small interfering RNA (siRNA), Lipofectamine RNAiMAX was used (Invitrogen, Thermo Fisher Scientific, Carlsbad, CA, USA) to transfect the following oligonucleotide pair sequences: si-Naa20 #1, sense 5′-GAUGAUUCUGGAGCUCUAU-3′ and antisense 5′-AUAGAGCUCCAGAAUCAUC-3′ (Bioneer, Daejeon, Korea); si-Naa20 #2, sense 5′-GCUAGGGAAAAGACUUGCU-3′ and antisense 5′-AGCAAGUCUUUUCCCUAGC-3′ (Bioneer, Daejeon, Korea); si-AMPKα, sense 5′-UGCCUACCAUCUCAUAAUAGAUAAC-3′ and antisense 5′-GUUAUCUAUUAUGAGAUGGUAGGCAAC-3′ (Integrated DNA technologies IDT, Iowa, USA); si-LBK1, 5′-CUGGUGGAUGUGUUAUACAACGAAG-3′ and antisense 5′-CUUCGUUGUAUAACACAUCCACCAGCU-3′ (IDT, Iowa, USA); and negative control, sense 5′-CGUUAAUCGCGUAUAAACGCGUAT-3′ and antisense 5′-AUACGCGUAUUAUACGCGAUUAACGAC-3′ (IDT, Iowa, USA). Transfections were conducted for 36 h according to the manufacturer’s instructions.

### Colony forming assay

Stable SK-Hep1 and Hep3B cells were plated in six-well plates (1 × 10^3^ cells/well) and grown in fresh medium at 37 °C for 10–12 days. Colonies were fixed with 80% methanol and stained with 0.5% crystal violet.

### Cell viability assay

CellTiter 96® AQueous One Solution Cell Proliferation assay (Promega, Madison, WI, USA) was performed to evaluate cell viability. Stable SK-Hep1 and Hep3B cell lines were plated in 96-well plates (1000 cells/well). Furthermore, 20 μL of MTS (3-(4,5-dimethylthiazol-2-yl)-5-(3-carboxymethoxyphenyl)-2-(4-sulfophenyl)-2H-tetrazolium) solution reagent was added to each well, and the plates were incubated at 37 °C for 1–4 h in a humidified atmosphere with 5% CO_2_. The plates were read in a 96-well plate reader at 490 nm.

### Cell proliferation assay

Stable SK-Hep1 and Hep3B cells were plated in 35-mm dishes (2 × 10^4^ cells/dish). Cells were harvested every 24 h after transfection. Then, 10-μL aliquots of the cell suspensions were measured using a hemocytometer.

### Western blot analysis and antibodies

Cells were lysed in RIPA buffer (50 mM Tris (pH 8.0), 150 mM NaCl, 0.1% SDS (sodium dodecyl sulfate), 0.5% Na-deoxycholate, and 1% NP-40) containing phosphatase and protease inhibitors. Then, cell lysates were obtained from the supernatant after centrifugation at 13,000 r.p.m. for 20 min. Protein concentrations were quantified using the Bradford assay. Protein samples were mixed with sample buffer (0.16 M Tris (pH 6.8), 12.5% β-mercaptoethanol, 12.5% SDS, 25% glycerol, and 0.05% bromophenol blue) in a boiling water bath for 5 min at 95 °C. Protein samples were separated by 10% or 12% SDS–polyacrylamide gel electrophoresis and transferred to PVDF membranes (Merck Millipore, Burlington, MA, USA). Membranes were incubated with primary antibodies specific for various proteins, including p-AMPKα (T172), AMPKα, p-mTOR (S2448), mTOR, p-4EBP1 (S65), 4EBP1, p-p70S6K (T389), p70S6K, β-actin, p-LKB1 (S428), LKB1, LC3B (all from Cell Signaling or Santa Cruz, CA, USA), Naa20 (Abclonal, Woburn, MA, USA), and p62 (Abcam, Cambridge, UK), at 4 °C overnight. After the membranes were washed three times for 5 min with PBST and incubated with secondary antibodies for 1 h at room temperature, they were washed again in PBST. Immunoreactive bands on the membranes were detected using ECL solution (Merck Millipore, Burlington, MA, USA).

### IP assay

Hep3B cells were lysed in immunoprecipitation (IP) buffer (20 mM HEPES (pH 7.0), 180 mM KCl, 0.2 mM EGTA, 1.5 mM MgCl_2_, 20% glycerol, and 1% NP-40), and 1 μg of the protein lysate was mixed with 50 μg of protein G agarose and the appropriate primary antibody. The IP mixture was incubated at 4 °C overnight on a rotator. The immunoprecipitated proteins were washed with IP wash buffer (20 mM HEPES (pH 7.0), 180 mM KCl, 0.2 mM EGTA, 1.5 mM MgCl_2_, 20% glycerol, and 0.1% NP-40) and eluted with loading buffer for western blot analysis.

### DTNB in vitro Nt-acetylation assay

An in vitro NAT activity assay was performed using Ellman’s reagent (5,5′-dithiobis-[2-nitrobenzoic acid] or DTNB), as previously described^25^. For purification of the NatB complex, HEK293T cells were transfected with V5-Naa20 WT or V5-Naa20 YF, lysed in IP buffer and immunoprecipitated using V5 beads. After determining by immunoblotting whether Naa25, which is an auxiliary subunit of the NatB complex, existed within the IPed V5-Naa20 WT or YF precipitate, we used it as the enzyme for this assay. Briefly, the immunoprecipitated NatB complex was incubated with 500 μM LKB1 substrate peptides and 500 μM acetyl-CoA in an acetylation buffer (100 mM HEPES-HCl (pH 7.5), 200 mM NaCl, and 2 mM EDTA) at 37 °C. After 30 min, the reaction was stopped by the addition of quenching buffer (3.2 M guanidinium-HCl and 100 mM sodium phosphate dibasic (pH 6.8)), mixed with DTNB buffer (100 mM sodium phosphate dibasic (pH 6.8), 10 mM EDTA, and 10 mg/mL DTNB), transferred to a 96-well plate, and spectrophotometrically analyzed at 412 nm. The absorbance, which was averaged from triplicate wells, was calculated for quantification of acetylation, as previously mentioned^25^. Peptides were custom-produced (GL Biochem, Shanghai, China) with a purity of 90–95%. The peptide abbreviations and sequences were as follows: LKB1 wild-type (WT), MEVVDPQQLGMFTEGE; Ac-LKB1, Ac-MEVVDPQQLGMFTEGE; LKB1-E2V, MVVVDPQQLGMFTEGE; and LKB1-MPE, MPEVVDPQQLGMFTEGE.

### LC–MS/MS analysis

Protein samples were processed with Thermo Scientific Pierce Detergent Removal Resin to remove any residual NP-40. Then, we used Amicon Ultra-0.5 mL 10-kDa centrifugal filters to remove the Flag peptide and to exchange the buffer from PBS to 0.1% RapiGest and 50 mM Tris-HCl (pH 8.0). The protein concentration was then determined by a bicinchoninic acid protein assay (Thermo Fisher Scientific, Carlsbad, CA, USA). Proteins in the sample were subsequently reduced and alkylated with 5 mM dithiothreitol at 45 °C for 30 min and 15 mM iodoacetamide at 45 °C in the dark for 30 min, respectively. Then, we digested the protein samples using sequencing-grade trypsin (Promega, Madison, WI, USA) at a ratio of 1:50 (micrograms of enzyme:micrograms of protein) at 37 °C overnight. After digestion, the sample was acidified using 1% TFA, incubated at 37 °C for 15 min to cleave the RapiGest surfactant, and centrifuged at 20,000 × *g* for 15 min. We transferred only the supernatant into a new tube, and the peptide sample was then purified, as previously described^26^. Briefly, the peptide sample was desalted over a C18 column and eluted in 0.1% formic acid in 60% acetonitrile. The eluted peptide sample was dried via vacuum centrifugation. The dried peptide sample was reconstituted with 10 µL of 0.1% formic acid.

Liquid chromatography-tandem mass spectrometry (LC–MS/MS) analysis was performed, as previously described^27^. Mass spectrometry data were acquired with an LTQ-Orbitrap XL mass spectrometer (Thermo Fisher Scientific, Carlsbad, CA, USA) coupled to an Eksigent nanoLC 2D LC system. In this system, purified peptides were separated on a microcapillary column (15 cm × 75 µm I.D.; packed in house with ReproSil Gold 120 C18, 5 µm resin (Dr. Maisch GmbH)) at a flow rate of 300 nL/min. Peptides were eluted with a linear gradient from 5 to 40% buffer B (0.1% formic acid in acetonitrile) over 90 min and 40 to 70% buffer B over 15 min. This elution step was followed by re-equilibration of the column with 5% buffer B for 15 min. Therefore, the total run time was 120 min. The eluted peptides were ionized under a spray voltage of 1.9 kV. The mass spectrometer was operated in data-dependent acquisition mode. In each data collection cycle, one full survey scan (300–2000 *m*/*z*) was acquired in the Orbitrap at a resolution of 60,000. Then, the top ten most abundant ions were selected for fragmentation by collision-induced dissociation in the ion trap with a precursor isolation window width of 2 *m*/*z*, an AGC setting of 1e^5^, and a maximum ion injection time of 500 ms.

The acquired data were searched against the UniProt Human reference database (released in June 2020) using SEQUEST (Proteome Discoverer 2.2), with a target-decoy strategy. The search parameters included a precursor mass tolerance of 20 p.p.m., a fragment ion tolerance of 0.6 Da and trypsin digestion with up to three missed cleavages. Carbamidomethylation of cysteine residues (+57.02146 Da) was set as the static modification, and methionine residue oxidation (+15.9949 Da) and protein Nt-acetylation (+42.0106 Da) were set as the dynamic modifications. To validate the peptide identifications, a peptide-level false discovery rate of <1% was used as a threshold. The annotated spectrum figure was generated using open-access PDV software.

### Analysis of TCGA-LIHC and GEO data

To compare the difference in Naa20 expression between HCC and normal tissues, publicly available data were used, including three HCC microarray data sets and data from a TCGA-LIHC (The Cancer Genome Atlas-Liver Hepatocellular Carcinoma) study. We retrieved the normalized expression data sets (GSE36411, GSE36376, and GSE54236) from the NCBI Gene Expression Omnibus (GEO) using the *GEOquery* R package. RNA-Seq gene expression data (Illumina HiSeq, FPKM normalization) from the TCGA-LIHC study were downloaded using the R/Bioconductor tool GenomicDataCommons (https://gdc.cancer.gov/), and the expression values were log2 transformed. Statistically significant differences were identified using the Wilcox test, and *P* values were adjusted using the Benjamini–Hochberg procedure.

### Statistical analysis

Data are expressed as the mean ± SEM of three or more independent experiments. Significant differences between groups were analyzed using Student’s *t* test. Statistical significance was accepted at *P < 0.05 and **P < 0.01.

## Results

### Naa20 is upregulated in tumors of HCC patients and promotes proliferation in HCC cell lines

**Fig. 1:**
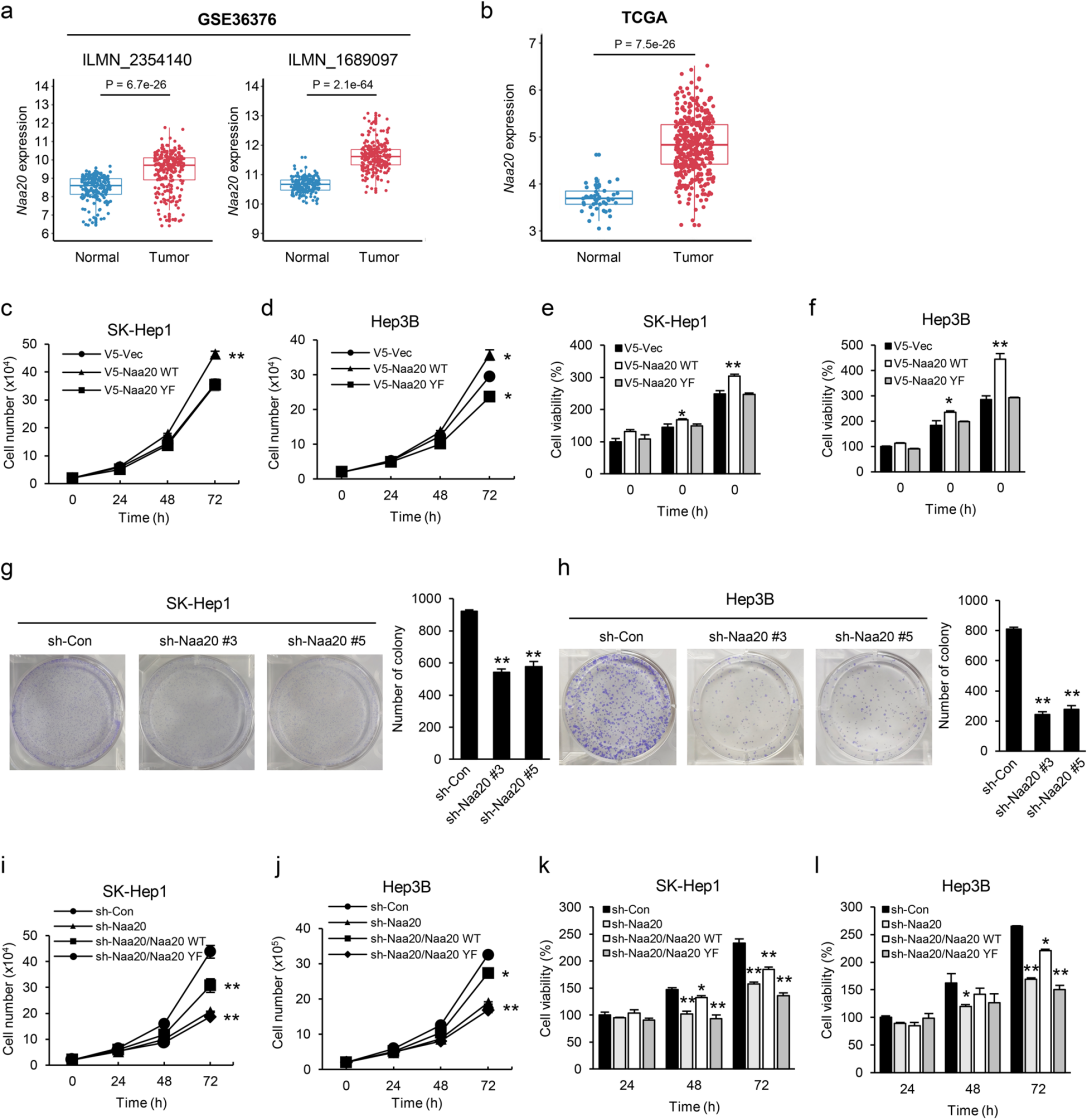
Naa20 is upregulated in HCC tumors and enhances oncogenic properties in HCC cell lines. **a**, **b** Naa20 mRNA expression levels in HCC tissues and adjacent normal tissues from the GEO data set GSE36376 (193 normal and 240 tumor tissues) (**a**) and TCGA-LIHC data (50 normal and 374 tumor tissues) (**b**) were analyzed, as described in the “Materials and methods” section. **c**–**f** SK-Hep1 (**c**, **e**) and Hep3B (**d**, **f**) cells were transiently transfected with V5-Naa20 WT or YF, followed by cell counting (**c**–**d**) and MTS assays (**e**–**f**) at the indicated times to determine the cell proliferation rates and cell viability, respectively. **g**–**h** Naa20 was stably silenced by a lentiviral system (sh-Naa20 #3 or #5) in SK-Hep1 (**g**) and Hep3B (**h**) cells, which were grown for 12 d, and were subsequently stained and counted. **i**–**l** V5-Naa20 WT or YF was reexpressed in Naa20-depleted SK-Hep1 (**i**, **k**) and Hep3B (**j**, **l**) cells, and cell counting (**i**–**j**) and MTS assays (**k**–**l**) were conducted at the indicated times to determine the cell proliferation rates and cell viability, respectively. All data are presented as the mean ± SEM of three independent experiments. *P < 0.05, **P < 0.01.

According to previous studies, the expression level of Naa20 is higher in HCC tumors than in nontumor tissues, and Naa20 silencing leads to retarded cell growth in HCC cell lines^11,12^, suggesting that Naa20 may act as an oncogenic factor in tumorigenesis. To further validate the clinical relevance of Naa20 in HCC patients, we analyzed microarray data from patients with HCC in GEO data sets and RNA-Seq data from the TCGA-LIHC data set. Bioinformatic analysis of several GEO data sets revealed that Naa20 expression levels were markedly higher in HCC tumors (GSE36411, *n* = 42; GSE36376, *n* = 240; and GSE54236, *n* = 81) than in nontumor tissues (GSE36411, *n* = 21; GSE36376, *n* = 193; and GSE54236, *n* = 80; Fig. 1a and Supplementary Fig. S1a, b). Consistent with this finding, bioinformatic analysis of TCGA-LIHC data also showed that Naa20 expression levels in tumors from HCC patients (*n* = 374) were significantly higher than those in normal tissues (*n* = 50; Fig. 1b). This analysis further indicates that Naa20 may be implicated in promoting HCC tumor progression.

Next, to explore the role of Naa20 on tumorigenic features in several HCC cell lines, including Hep3B, SK-Hep1, and HepG2, cells were transiently transfected with expression vectors of wild-type human Naa20 or the catalytically inactive mutant Naa20 YF (Y123F; Supplementary Fig. S2a, b)^28^, followed by cell counting and MTS assays to determine the cell growth rate and cell viability, respectively, at different times. Overexpression of Naa20 WT but not Naa20 YF led to increased cell growth and viability compared with that in mock-control in SK-Hep1 or Hep3B cells (Fig. 1c–f), indicating that Naa20 may promote tumorigenesis through its enzymatic activity. To further support this finding, Naa20 was silenced in cells by lentiviral transduction of two different sh-Naa20 vectors (#3 and #5) (Supplementary Fig. S2c, d), and colony formation, cell counting, and MTS assays were conducted to analyze tumorigenic features. In agreement with previous reports^11,12^, Naa20 depletion significantly reduced cell proliferation and viability compared with that in control cells (Fig. 1g–l). Importantly, reexpression of Naa20 WT in Naa20-silenced cells (Supplementary Fig. S2e, f) rescued these phenotypes, but expression of the catalytically inactive mutant Naa20 YF did not (Fig. 1i–l), further validating the oncogenic role of Naa20 in tumorigenesis.

### Naa20 deficiency stimulates AMPK activity to suppress the mTOR signaling pathway

**Fig. 2:**
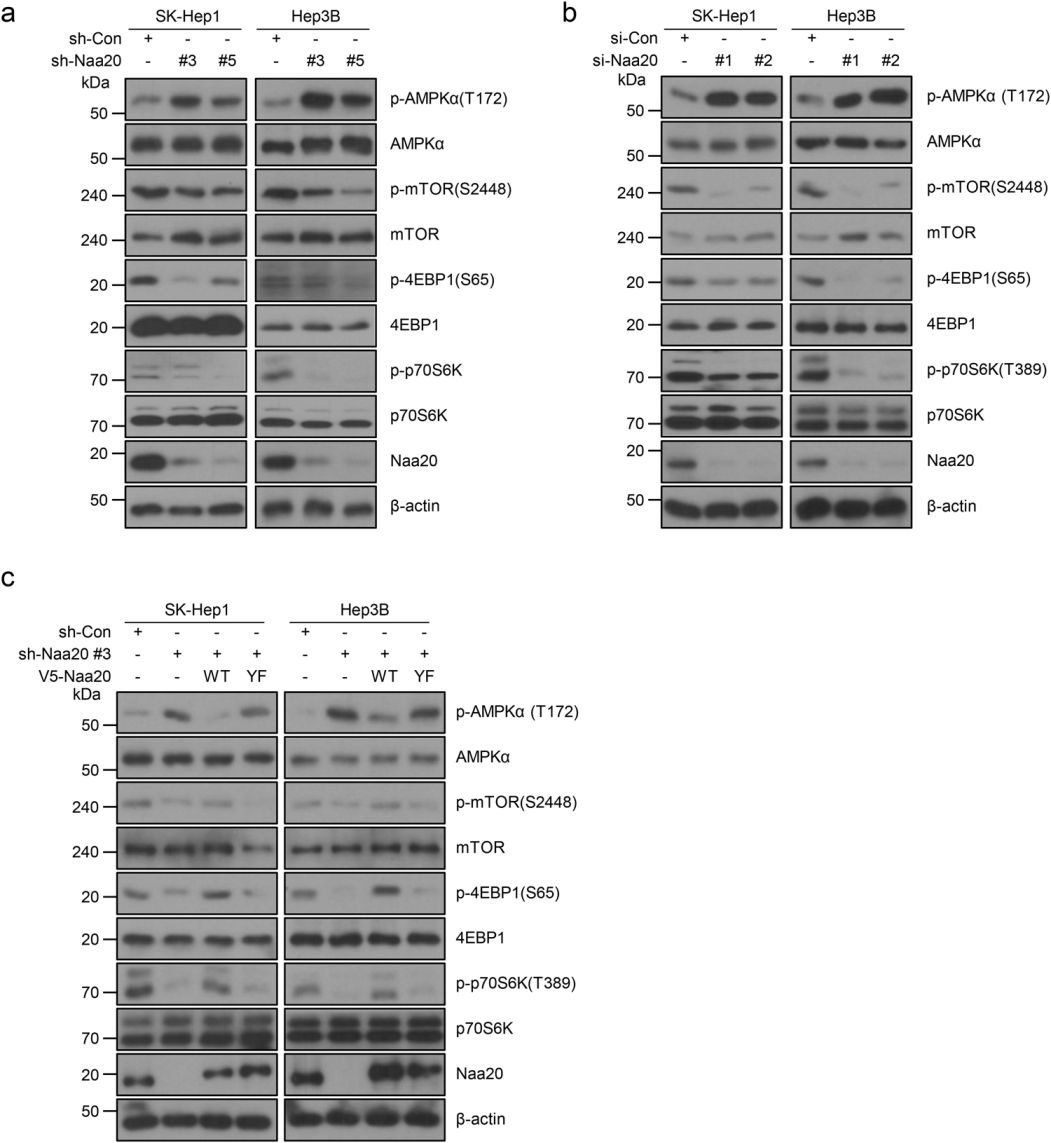
Naa20 deficiency activates AMPK to promote the mTOR signaling pathway. **a** Naa20 was stably knocked down with the lentiviral system (sh-Naa20 #3 or #5) in SK-Hep1 and Hep3B cells, as analyzed by western blotting using the indicated antibodies. **b** Naa20 was transiently silenced by transfection of si-Naa20 #1 or #2 into SK-Hep1 and Hep3B cells, and western blot analysis was then performed using the indicated antibodies. **c** V5-Naa20 WT or YF was reexpressed in SK-Hep1 and Hep3B cells with stable Naa20 silencing, and western blot analysis was then conducted using the indicated antibodies.

According to a previous report^23^, the naa20-Δ deletion mutant in yeast displayed increased phosphorylation levels, and the kinase Snf1p, the yeast homolog of AMPK, was most prominently involved in this process. Moreover, recent studies showed that silencing Naa20 in cells led to increased autophagy^14,15^. Because the AMPK–mTOR signaling pathway has been well documented to play an important role in autophagy and cell growth in mammals^24,29^, we investigated whether the Naa20 level is correlated with AMPK–mTOR signaling pathway activity. First, we examined the expression and phosphorylation (T172) levels of AMPKα in HCC cell lines transfected with sh-con and sh-Naa20. As expected, Naa20 depletion led to enhanced phosphorylation levels of AMPK (T172; Fig. 2a). Since AMPK has been well reported to be a major inhibitor of mTOR^24^, we next investigated the mTOR signaling pathway by monitoring the phosphorylation of mTOR at S2448, as well as the phosphorylation of the well characterized mTOR downstream molecules S6K (ribosomal protein S6 kinase) at T389 and 4E-BP (eukaryotic translation initiation factor 4E-binding protein) at S65 (ref. ^24^). Consistent with the above finding, Naa20-silenced cells also displayed a marked reduction in mTOR signaling compared with that in control cells (Fig. 2a). To exclude the possibility that this reduction might result from long-term adaption of Naa20-depleted cells, endogenous Naa20 was transiently depleted in HCC cells by RNA interference (RNAi), followed by western blot analysis for investigation of AMPKα and mTOR levels. Consistent with the previous finding, these results showed that Naa20 deficiency led to not only greatly increased p-AMPK levels, but also markedly reduced mTOR signaling in all HCC cell lines analyzed (Fig. 2b and Supplementary Fig. S3). Importantly, reconstitution of Naa20 WT, but not Naa20 YF, in Naa20-depleted cells sufficiently reversed the molecular events caused by Naa20 deficiency (Fig. 2c), further indicating that Naa20 may regulate the AMPK–mTOR axis through its catalytic activity.

### Naa20 contributes to cell growth and autophagy by regulating AMPK activity

**Fig. 3:**
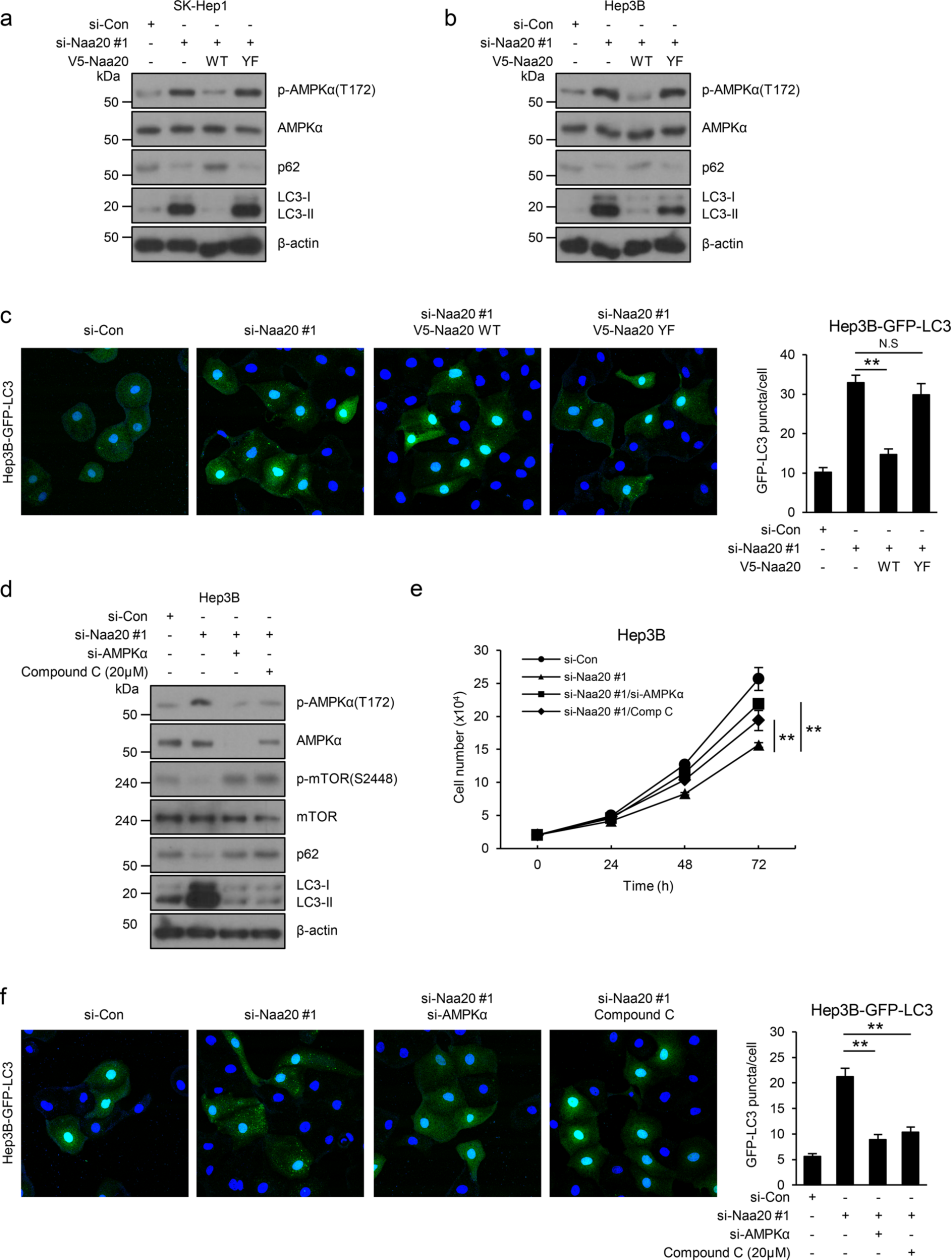
Loss of Naa20 promotes autophagy and cell proliferation through AMPK-dependent inhibition of the mTOR signaling pathway. **a**, **b** Naa20-silenced SK-Hep1 (**a**) or Hep3B (**b**) cells were transfected with V5-Naa20 WT or YF, and western blot analysis was then performed using the indicated antibodies. **c** V5-Naa20 WT or YF was reexpressed in Hep3B-GFP-LC3 cells with stable Naa20 silencing, and fluorescence microscopy analysis was conducted for quantification of GFP-LC3B puncta. **d**–**f** For genetic or pharmacologic inhibition of Naa20, Hep3B (**d**, **e**), or Hep3B-GFP-LC3 (**f**) cells were cotransfected with si-Naa20 #1 and si-AMPKα or transfected with only si-Naa20 #1, and then treated with compound C (20 μM) followed by western blot analysis (**d**), cell counting (**e**) and fluorescence microscopy analysis (**f**). All data are presented as the mean ± SEM of three independent experiments. *P < 0.05, **P < 0.01. All GFP-LC3B puncta quantification data were analyzed with Zeiss LSM880 Airyscan microscopes at Ewha Fluorescence Core Imaging Center, Ewha Womans University.

The AMPK–mTOR axis reportedly plays a critical role in autophagy^24^, and previous studies showed that Naa20 knockdown resulted in increased autophagy activation^14,15^. Hence, we next investigated whether Naa20 might be involved in autophagy activation. To this end, Naa20 was knocked down by RNAi in Hep3B and SK-Hep1 cells, and V5-Naa20 WT or YF was reexpressed in these cells followed by western blot analysis using antibodies against autophagy markers, such as p62 and LC3. Indeed, increased levels of LC3-II and reduced levels of p62 were detected in extracts of Naa20-deficient cells compared with control cells, and the original levels were restored by reconstitution of Naa20 WT but not Naa20 YF (Fig. 3a, b), indicating that Naa20 regulates autophagy. Consistent with this finding, silencing of Naa20 in cells stably expressing GFP-LC3 also led to greatly increased GFP-LC3 distribution in cytoplasmic puncta (Fig. 3c). Collectively, these data indicate that Naa20 regulates autophagy in an Nt-acetylation-dependent manner.

Next, to determine whether AMPK plays a critical role in the growth retardation and autophagy upregulation driven by Naa20 deficiency, Naa20-deficient Hep3B cells were treated with the AMPK inhibitor compound C or transiently transfected with siRNA specific for AMPKα, followed by western blot, cell count, and autophagy marker analysis. Western blot analysis revealed that AMPK inhibition reversed the alterations in p-mTOR (S2448) and autophagic marker levels elicited by Naa20 depletion in Hep3B cells (Fig. 3d). Furthermore, AMPK inhibition was sufficient to rescue the delayed cell growth, as well as the increased autophagy observed in Naa20-depleted cells (Fig. 3e, f). Collectively, these results demonstrate that AMPK may be essential for the phenotypes observed in Naa20-deficient cells.

### Nt-acetylation by Naa20 is implicated in LKB1 activity toward AMPK

**Fig. 4:**
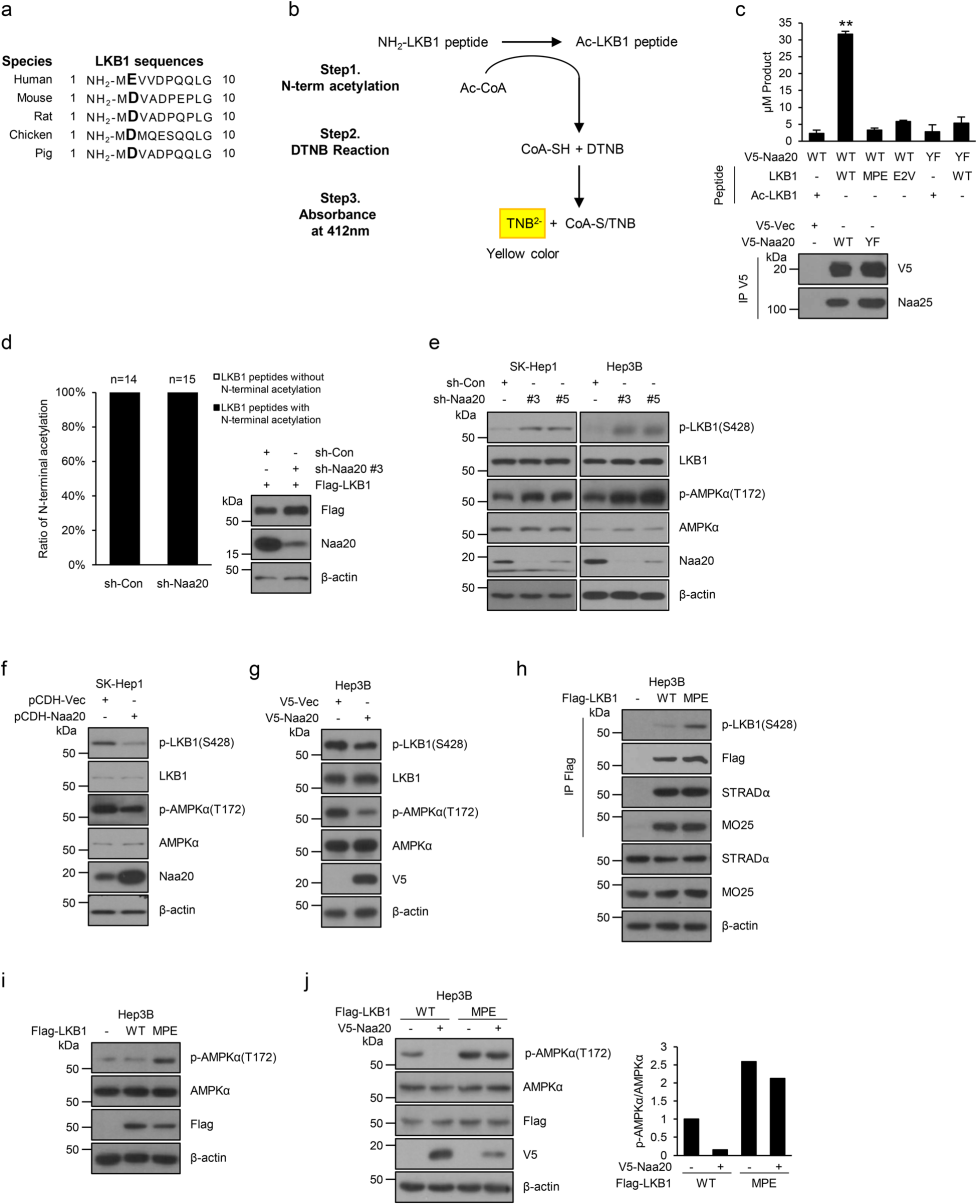
Naa20 acetylates the N-terminus of LKB1 in vitro and reduces its activity toward AMPK. **a** Sequence alignment of LKB1 N-termini from several species. **b** Scheme of the DTNB-based in vitro Nt-acetylation assay. **c** To determine whether LKB1 is a substrate of the NatB complex, a DTNB-based in vitro Nt-acetylation assay was performed as described in the “Materials and methods” section. Ac-LKB1, LKB1-MPE, and LKB1-E2V peptides were used as negative control substrates; *P < 0.05, **P < 0.01. **d** Assessment of the N-terminal acetylation of LKB1. N-terminal acetylation of Flag-LKB1 overexpressed in sh-Con or sh-Naa20 HEK293T cells was analyzed by mass spectrometry. In the right panel, sh-Con or sh-Naa20 #3 HEK293T cells were transfected with Flag-LKB1, and western blot analysis was then conducted using the indicated antibodies. **e** SK-Hep1 and Hep3B cells were infected with lentiviruses expressing sh-Naa20 #3 or #5 and were then analyzed by western blotting using the indicated antibodies. **f**–**g** Wild-type Naa20 was overexpressed in SK-Hep1 (**f**) and Hep3B (**g**) cells, followed by western blot analysis using the indicated antibodies. **h** Flag-tagged Naa20 WT or MPE was overexpressed in Hep3B cells. Flag-Naa20 WT and MPE were immunoprecipitated separately with an anti-Flag antibody, followed by western blot analysis using the indicated antibodies. **i** Flag-LKB1 WT or MPE was overexpressed in Hep3B cells, followed by western blot analysis using the indicated antibodies. **j** Flag-LKB1 WT or MPE was overexpressed with or without V5-Naa20 in Hep3B cells, followed by western blot analysis using the indicated antibodies. The bands were quantified using image analysis software, and the relative band intensities were expressed as p-AMPKα/AMPKα ratios.

Next, we addressed the question of how the enzymatic function of Naa20 affects AMPK activity. Because it has been reported that the substrate specificity of NatB is determined by the first two N-terminal residues containing a methionine-acidic/hydrophilic amino acid motif, such as MD-, MN-, ME-, or MQ^1–4^, we focused on the N-terminal amino acids of AMPK or its regulators. Among these proteins, LKB1 contains Met-Glu (ME) and Met-Asp (MD) as the first two N-terminal residues in humans and other species, respectively (Fig. 4a), indicating that LKB1 may be a possible substrate of Naa20. This finding encouraged us to investigate whether LKB1 is subjected to Nt-acetylation by NatB. To first determine whether Naa20 directly Nt-acetylates LKB1 at the methionine residue of the N-terminus in vitro, we performed an in vitro Nt-acetylation assay using DTNB-based quantification, as indicated in Fig. 4b; this method is a simple, fast, and nonisotope method for in vitro quantification of Nt-acetylation^25^. The in vitro Nt-acetylation assay revealed that Naa20 WT significantly increased the Nt-acetylation of LKB1 wild-type peptides with nonacetylated methionine at the N-terminus, whereas it had no effect on the Nt-acetylation of two LKB1 mutant peptides with a NatB-permissive substitution or insertion at the second amino acid of E to V (LKB1-E2V), which is recognized by NatA but not NatB, or to P (LKB1-MPE), which should be unacetylated based on a previous report (Fig. 4c), respectively. However, the catalytically inactive Naa20 mutant had only minimal or no activity toward LKB1 WT (Fig. 4c), suggesting that Naa20 can directly Nt-acetylate LKB1 with high specificity. Next, to investigate whether Naa20-mediated Nt-acetylation of LKB1 occurs at the cellular level, LKB1 WT with a C-terminal Flag tag was expressed in HEK293T cells transduced with sh-con or sh-Naa20 lentiviral vectors, purified by IP with an anti-Flag antibody, and analyzed for N-terminal modification by nanoLC–MS/MS. Unexpectedly, the results showed that all detected N-terminal peptides of LKB1 were N-terminally acetylated not only in control cells but also in Naa20-deficient cells (Fig. 4d and Supplementary Fig. S4a, b), indicating that the N-terminus of LKB1 is indeed modified by Nt-acetylation, but this Nt-acetylation may be catalyzed by NatB, as well as other members of the NAT family. Furthermore, reciprocal coimmunoprecipitation analysis using exogenous or endogenous proteins revealed that Naa20 and LKB1 interact with each other (Supplementary Fig. S5a–c), indicating that Naa20 may be closely correlated with LKB1. Taking these results together, we presumed that Naa20 may play its role in modulating AMPK activity through Nt-acetylation of LKB1, although we could not exclude other possible mechanisms related or unrelated to LKB1 activity.

It has been known that LKB1 activity can be regulated by diverse ways such as through the formation of a complex with the pseudokinase STRADα, and the scaffolding protein MO25 and via PTMs, such as phosphorylation, ubiquitination, neddylation, and lysine acetylation^19,20^. To gain further insights into the role of Naa20 in modulating LKB1 activity, we first determined whether Naa20 regulates the protein stability or phosphorylation levels (S428) of LKB1. For this, Naa20 was silenced or overexpressed in SK-Hep1 and Hep3B cells, followed by immunoblot assay. Interestingly, p-LKB1 (S428) and p-AMPK levels were markedly elevated in Naa20-silenced cells and, in contrast, were greatly reduced in Naa20-overexpressing cells compared with control cells (Fig. 4e–g and Supplementary Fig. S6a, b), suggesting that Naa20 may negatively affect the phosphorylation level of LKB1 and its activity toward AMPK.

Next, to further validate whether Nt-acetylation of LKB1 regulates its activity toward AMPK through modulation of its phosphorylation, Flag-tagged LKB1 WT or LKB1-MPE mutant was expressed in Hep3B cells, which were subjected to IP with an anti-Flag antibody and western blot analysis with the antibodies indicated in Fig. 4h. Importantly, cells expressing LKB1-MPE, which should be not Nt-acetylated by NATs, showed significantly increased p-LKB1 levels compared with cells expressing LKB1 WT (Fig. 4h). However, there were no differences in the formation levels of the complexes containing LKB1 WT or LKB1-MPE and STRADα/MO25, a major regulator of LKB1 (Fig. 4h). These findings indicate that Nt-acetylation of LKB1 can inversely affect p-LKB1 levels by unknown mechanisms. Moreover, expression of the LKB1-MPE mutant led to notably increased p-AMPK levels compared with those in cells expressing LKB1 WT or LKB1-E2V, which is expected to be Nt-acetylated by NatA but not NatB (Fig. 4i). Most importantly, coexpression of Naa20 in cells transfected with LKB1 WT, but not in cells transfected with LKB1-MPE provoked an obvious reduction in the p-AMPK level compared with that in control cells (Fig. 4j). These results indicate that Naa20 may suppress LKB1 activity toward AMPK at least partially through Nt-acetylation of LKB1, although we cannot exclude other possible mechanisms unrelated to LKB1.

### Naa20 regulates cell growth and autophagy through the LKB1-mediated AMPK–mTOR signaling pathway

**Fig. 5:**
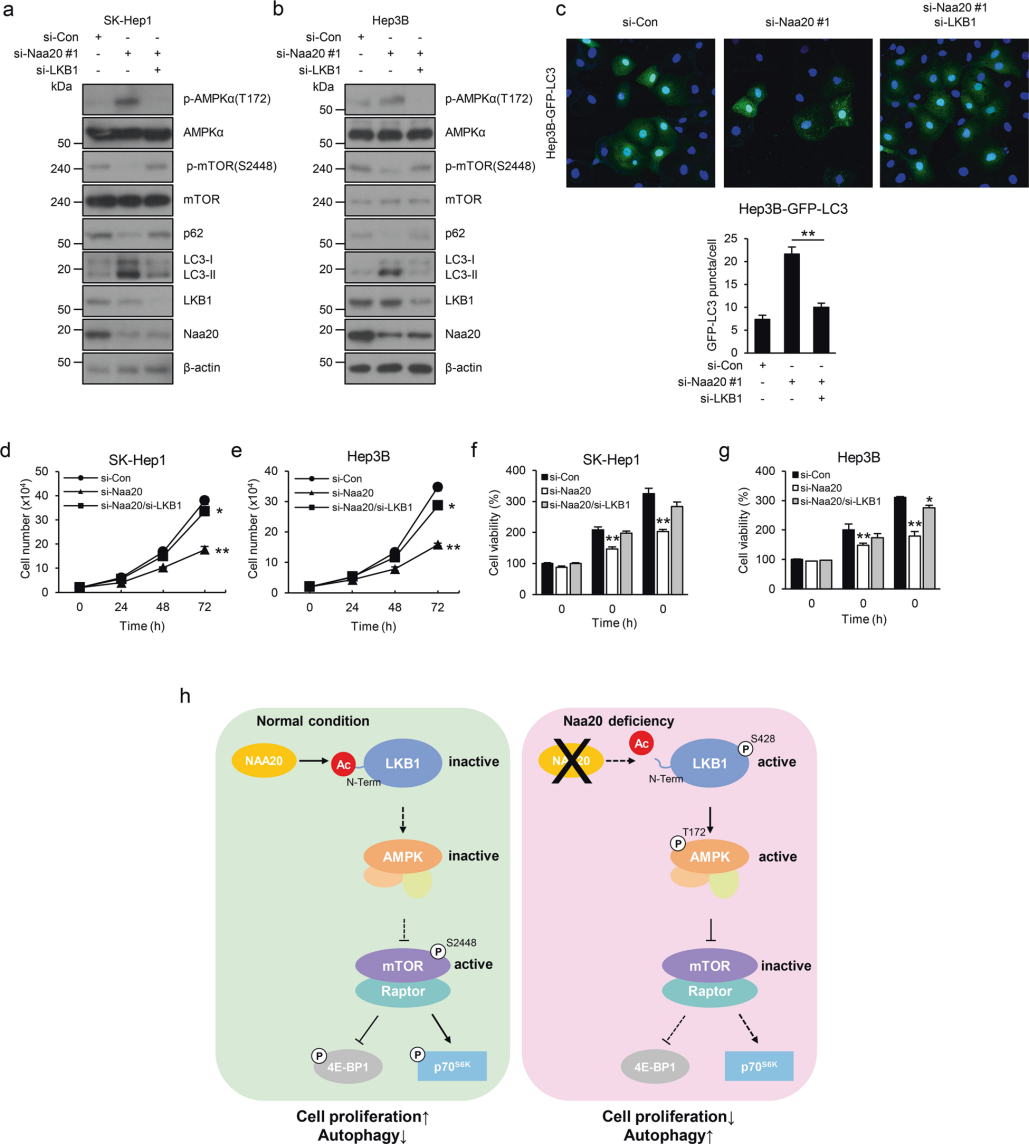
Naa20-mediated cell proliferation and autophagy are dependent on LKB1 in HCC cells. **a**–**g** si-Naa20 was transfected alone or cotransfected with si-LKB1 into SK-Hep1 (**a**, **d**, **f**), Hep3B (**b**, **e**, **g**), or stable Hep3B-GFP-LC3 (**c**) cells. Western blot analysis (**a**, **b**) was then conducted using the indicated antibodies; cell counting (**d**, **e**), and MTS assays (**f**, **g**) were performed to evaluate the cell proliferation rates or cell viability, respectively, and fluorescence microscopy analysis (**c**) was conducted for quantification of LC3B puncta. **h** Proposed model showing how Naa20 contributes to cell proliferation and autophagy through the LKB1–AMPK–mTOR signaling pathway in HCC cells. All data are presented as the mean ± SEM of three independent experiments. *P < 0.05, **P < 0.01.

To confirm our hypothesis that Naa20 regulates the AMPK–mTOR axis through LKB1, which contributes to cell growth and autophagy, Naa20 was transiently silenced by RNAi in HCC cell lines, and the AMPK–mTOR signaling pathway, cell proliferation rate, and autophagy activation levels were then assessed in these cell lines. In support of our findings, the results showed that LKB1 deficiency in Naa20-silenced cells reversed the alterations in the AMPK–mTOR signaling pathway caused by Naa20 depletion (Fig. 5a, b). Consistent with this result, LKB1 deficiency also reversed the cellular phenotypes, such as autophagy activation and enhanced cell growth, elicited by Naa20 depletion (Fig. 5c–g). Taken together, these results strongly indicate that LKB1 may play a crucial role in the growth retardation and autophagy activation observed in Naa20-deficient HCC cells (Fig. 5h).

## Discussion

The NatB complex has recently been reported to act as an oncogenic effector in HCC and to be involved in autophagy^11–15^. However, the molecular mechanism underlying the involvement of Naa20 in tumorigenesis and autophagy remains elusive. In this study, we provide evidence further supporting previous reports that Naa20 acts as an oncogenic factor, as well as an autophagy suppressor in HCC cell lines^11–15^ and, importantly, propose a novel and plausible mechanism responsible for that activity: Naa20 inhibits AMPK activity to promote the mTOR signaling pathway, which contributes to tumorigenesis and autophagy. Naa20-mediated inhibition of AMPK may be largely controlled by LKB1 activity. Importantly, LKB1 may undergo Nt-acetylation by NatB and/or, likely, other members of the NAT family in vitro and in vivo, which may negatively influence both its phosphorylation and activity toward AMPK (Fig. 5h).

Although previous studies have revealed the implication of Naa20 in tumorigenesis and autophagy in HCC^11–15^, the underlying mechanism remains unclear. In this respect, our data reported here show that Naa20 negatively regulates the phosphorylation (T172) and activity of AMPK to promote the mTOR signaling pathway in a Naa20 catalytic activity-dependent manner, leading to the induction of oncogenic features and suppression of autophagy in HCC cells. AMPK has been consistently reported to suppress oncogenic features and activate autophagy in several types of cancer, including HCC^24,29^. Notably, a recent study revealed that Naa20 depletion in yeast led to elevated protein phosphorylation, possibly owing to Snf1, the yeast homolog of AMPK^23^. Collectively, these results suggest that AMPK plays an important role in Naa20-mediated regulation of tumorigenesis and autophagy.

How does Naa20 regulate the activity of AMPK? The activity of AMPK is largely regulated by the ratio of cellular AMP or ADP to ATP or by phosphorylation (T172) mediated by several protein kinases or phosphatases, such as LKB1, calcium/calmodulin-dependent kinase kinase 2, protein phosphatase 2, or protein phosphatase 2C, in a cellular context- or cell type-dependent manner^24,29^. Among these proteins, LKB1 attracted our attention as one of the Naa20 substrates because it has an N-terminus that starts with a Met-Glu motif, one of the determinants of the substrate specificity of NatB. Consequently, we showed here that LKB1 is N-terminally acetylated within cells in vivo or by NatB in vitro. However, we also found that all N-terminal peptides of LKB1 detected in Naa20-silenced cells were still N-terminally acetylated, indicating that LKB1 may either not be a substrate of NatB or can be a substrate of other NATs. This discrepancy between the in vitro and in vivo results might have occurred because of the cell types used, the efficiency of Naa20 knockdown, or the existence of substrate redundancy among NATs. Indeed, a previous report showed that the successful identification of NatB substrates in cells could be largely affected by the knockdown efficiency or by substrate redundancy among NATs^4^. Although we could not determine whether LKB1 is a target of NatB in vivo, we propose, based on a previous report, that the acetylation status of LKB1 at its N-terminus can affect its activity toward AMPK. Indeed, overexpression of the LKB1 mutant (LKB1-MPE), which is not expected to be Nt-acetylated by NATs, led to greatly enhanced LKB1 and AMPK activity compared with that in cells with overexpression of wild-type LKB1. Furthermore, Naa20 significantly inhibited AMPK activity in cells expressing LKB1 WT, but only slightly in cells expressing the LKB1-MPE mutant. Importantly, knockdown of LKB1 in Naa20-deficient cells was sufficient to reverse the upregulation of AMPK driven by Naa20 deletion. Overall, these data support our hypothesis that Naa20-mediated regulation of AMPK can result at least partially from Nt-acetylation of LKB1 by Naa20. In addition, a recent report^9,10^ noting that yeast cells lacking NatB exhibit significantly reduced NAD^+^ levels suggests another convincing hypothesis, because a marked reduction in the NAD^+^ level subsequently results in ATP depletion and promotion of AMPK activity^30,31^. Hence, this possibility should be considered in future studies.

Although LKB1 has been well documented as a tumor suppressor in a variety of cancers^19^, it has also been shown to have an ambivalent role in HCC tumorigenesis^20,21,32^. For example, several liver tumors from mice or HCC tumors from patients with poor prognosis were found to have the highest levels of phosphorylated LKB1 (S428)^20,21,33^, indicating that phosphorylation of LKB1 at Ser428 may be involved in promoting tumorigenesis. However, we showed here that LKB1 may function as a tumor suppressor in tumorigenesis in a manner related to Naa20 in HCC cells. Indeed, accumulating evidence indicates that the role of LKB1 phosphorylation (S428) in HCC tumorigenesis seems to be partly contradictory depending on the cell type and cellular context^20,21,33^. Moreover, recent reports have revealed that LKB1 activity in HCC might be correlated with diverse types of posttranslational modifications, such as phosphorylation, neddylation, ubiquitination, and acetylation^19,20^, indicating that detailed analysis of PTMs in the LKB1 protein might be helpful for determining the role of LKB1 in HCC. Thus, to understand the dual role of LKB1 in HCC, more research should focus on the involvement of PTMs, as well as intermediates of the LKB1 signaling pathway in HCC.

An interesting question is how Nt-acetylation of LKB1 affects its own phosphorylation. Our data suggest that Nt-acetylation may participate in crosstalk with phosphorylation of the LKB1 protein. Many studies have revealed that Nt-acetylation can have various effects on proteins^1–3^. One effect is that Nt-acetylation can participate in crosstalk with other types of modification at the N-terminus or in internal regions within the same protein^1–3^. For example, NatD-mediated histone H4 Nt-acetylation inhibits H4Arg3 methylation and/or H4Ser1 phosphorylation, leading to alterations in some gene transcription programs^34,35^. Therefore, how the Nt-acetylation of LKB1 affects its phosphorylation of LKB1 is an interesting topic for future studies.

Overall, this study reveals a novel mechanism by which Naa20 contributes to tumorigenic behaviors and autophagy in HCC cells, and thereby sheds light on the potential therapeutic targeting of Naa20 in HCC.

## Supplementary information

Supplementary information
