# Supplementary material for: Naa20, the catalytic subunit of NatB complex, contributes to hepatocellular carcinoma by regulating the LKB1–AMPK–mTOR axis
Source: Exp Mol Med. 2020 Nov 20;52(11):1831–44. doi: 10.1038/s12276-020-00525-3 (PMC8080711; doi:10.1038/s12276-020-00525-3)
Supplement: Supplementary file 1 — Supplementary information [file 12276_2020_525_MOESM1_ESM.pdf]

Supplementary Figure S1. Naa20 expression levels were higher in tumor tissues of HCC patients than that in normal tissues.

To compare Naa20 expression levels between non-tumor and tumor tissues from HCC cancer patients, (a–b) bioinformatic analysis of the GEO data sets, GSE36411 (21 normal and 42 tumor tissues) (a) and GSE54236 (80 normal and 81 tumor tissues) (b) was performed as described in the Material and Methods.

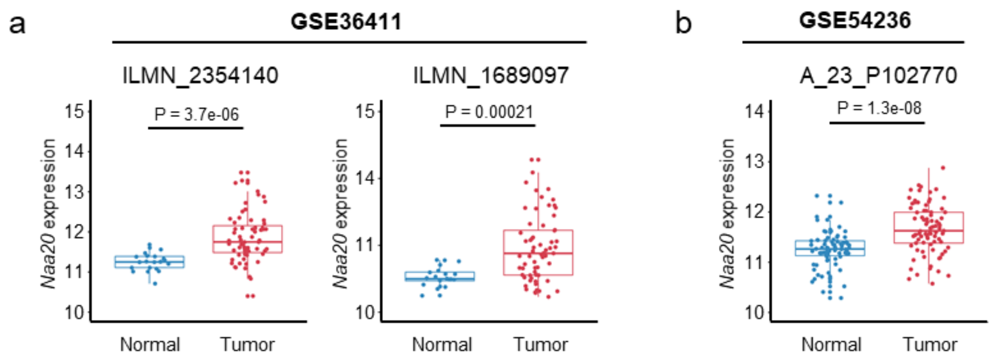

Jung et al., Sup Fig1

Supplementary Figure S2. Over-expression and depletion of Naa20 in HCC cell lines.

(a–b) SK-Hep1 (a) and Hep3B (b) cells were overexpressed with V5-Naa20 WT or YF. (c–d) Naa20 was silenced by a lentiviral system (sh-Naa20 #3 or #5) in SK-Hep1 (c) and Hep3B (d) cells. (e–f) Naa20-deficient SK-Hep1 (e) or Hep3B (f) stable cell lines were restored with V5-Naa20 WT or YF. All were followed by Western blot analysis using antibodies as indicated.

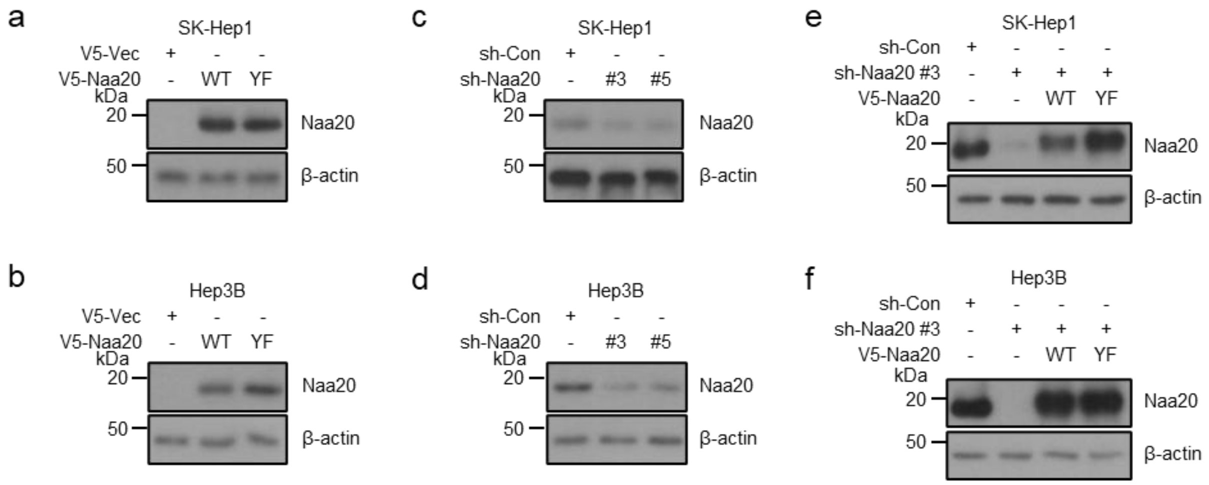

Jung et al., Sup Fig2

Supplementary Figure S3. Depletion of Naa20 activates AMPK activity toward mTOR signaling pathway in HepG2 cells.

Naa20 was silenced by RNAi, si-Naa20 #1 or #2 in HepG2 cells, which were followed by Western blot analysis using antibodies as indicated.

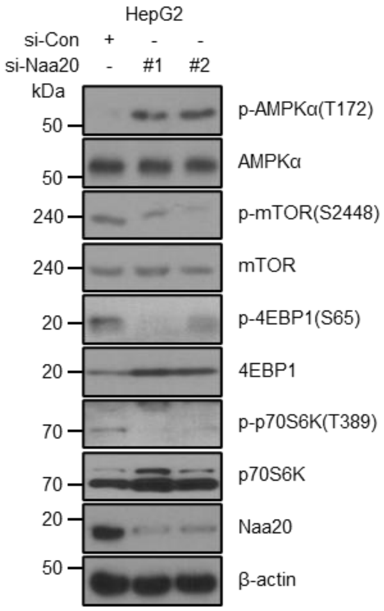

Jung et al., Sup Fig3

Supplementary Figure S4. LKB1 is N-terminally acetylated in HEK293T cells.

(a–b) LKB1 acetylated in the sh-Con or sh-Naa20 cell lines was digested with trypsin and the HPLC fraction containing the peptide was analyzed by LC-MS/MS.

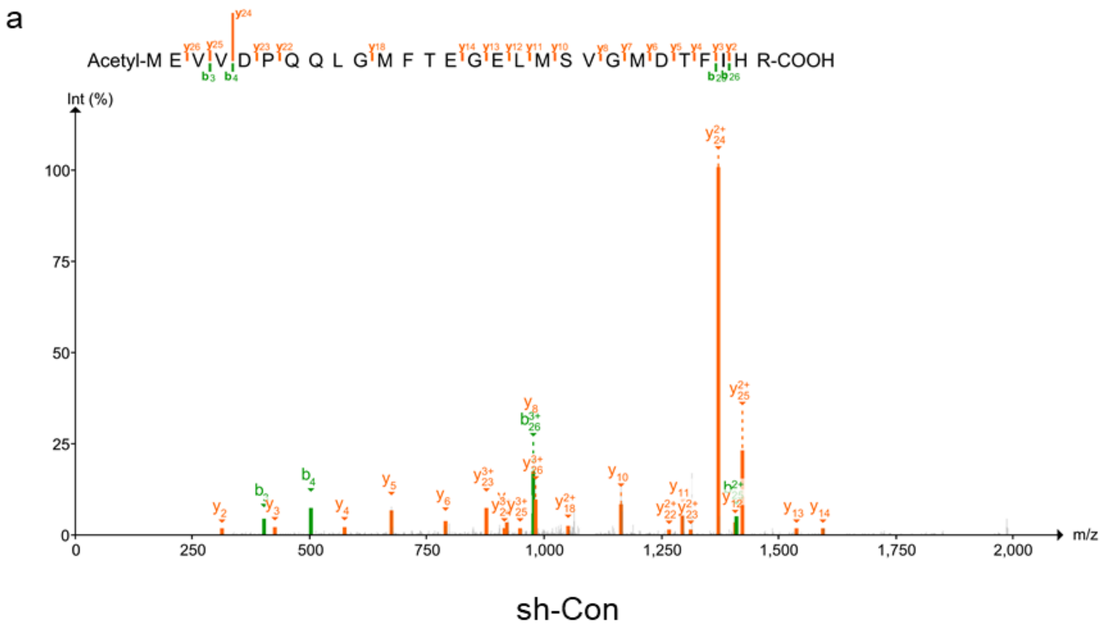

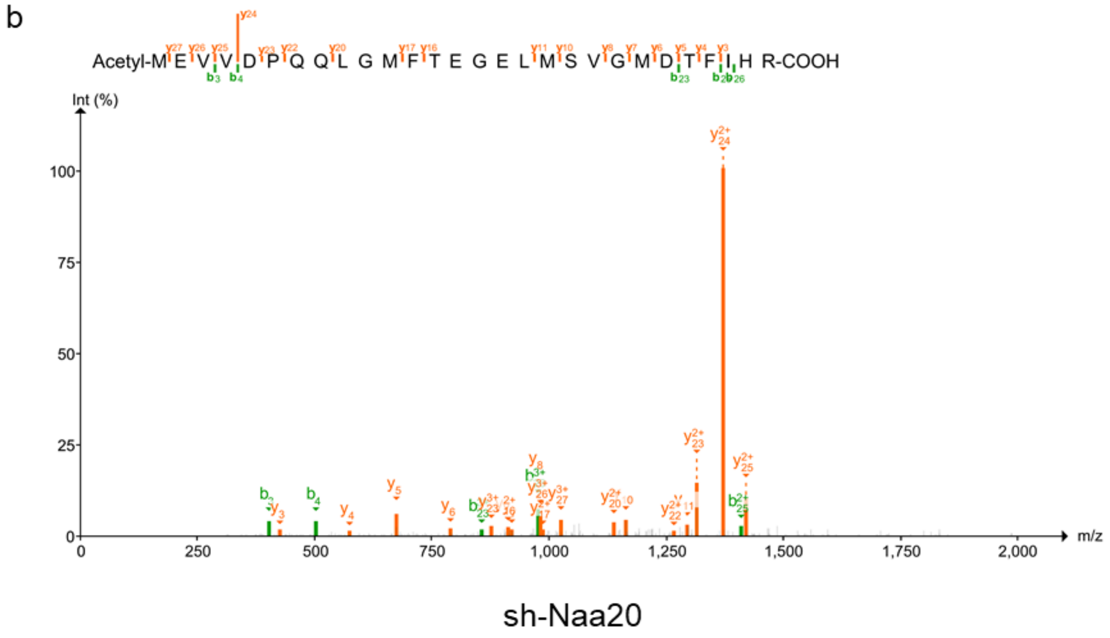

Jung et al., Sup Fig4

Supplementary Figure S5. Naa20 interacts with LKB1.

(a–c) For reciprocal interaction analysis between LKB1 and Naa20, either Flag-LKB1 or V5-Naa20 was transfected in Hep3B cell lines, which was immunoprecipitated with Flag (a) or V5 (b) antibody, respectively, followed by Western blot analysis using the indicated antibodies. (c) Endogenous LKB1 or Naa20 protein in Hep3B cells was immunoprecipitated and immunoblotted with the indicated antibodies.

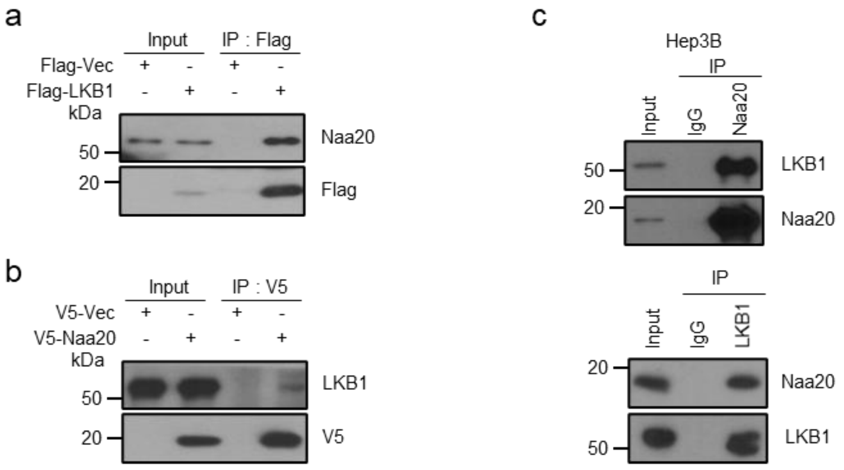

Jung et al., Sup Fig5

Supplementary Figure S6. Naa20 modulates LKB1-AMPK in HepG2.

(a–b) Naa20 was silenced by sh-Naa20 #3 or #5 lentiviral system (a) and over-expressed by transfection with Naa20 expression vector (pCDH-Naa20) (b) in HepG2 cells, which were analyzed by Western blot assay using antibodies as indicated.

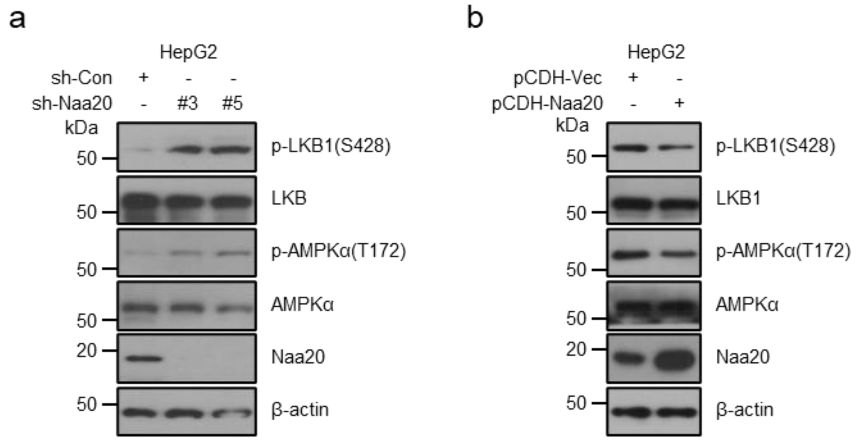

Jung et al., Sup Fig6
